# Environment-Friendly Catalytic Mineralization of Phenol and Chlorophenols with Cu- and Fe- Tetrakis(4-aminophenyl)-porphyrin—Silica Hybrid Aerogels

**DOI:** 10.3390/gels8040202

**Published:** 2022-03-23

**Authors:** Enikő Győri, Ádám Kecskeméti, István Fábián, Máté Szarka, István Lázár

**Affiliations:** 1 Department of Inorganic and Analytical Chemistry, University of Debrecen, Egyetem tér 1, H-4032 Debrecen, Hungary; gyori.eniko@science.unideb.hu (E.G.); adam.kecskemeti@teva.hu (Á.K.); ifabian@science.unideb.hu (I.F.); 2 MTA-DE Redox and Homogeneous Catalytic Reaction Mechanisms Research Group, University of Debrecen, Debrecen Egyetem tér 1, H-4032 Debrecen, Hungary; 3 Institute for Nuclear Research, Debrecen Bem tér 18/c, H-4026 Debrecen, Hungary; szarka.mate@atomki.hu; 4 Vitrolink Kft., Debrecen, Sárosi utca 9, H-4033 Debrecen, Hungary

**Keywords:** silica aerogel, aerogel hybrid, covalent immobilization, porphyrin complexes, heterogeneous catalyst, phenol mineralization, chlorophenol mineralization

## Abstract

Fenton reactions with metal complexes of substituted porphyrins and hydrogen peroxide are useful tools for the mineralization of environmentally dangerous substances. In the homogeneous phase, autooxidation of the prophyrin ring may also occur. Covalent binding of porphyrins to a solid support may increase the lifetime of the catalysts and might change its activity. In this study, highly water-insoluble copper and iron complexes of 5,10,15,20-tetrakis(4-aminophenyl)porphyrin were synthesized and bonded covalently to a very hydrophilic silica aerogel matrix prepared by co-gelation of the propyl triethoxysilyl-functionalized porphyrin complex precursors with tetramethoxysilane, followed by a supercritical carbon dioxide drying. In contrast to the insoluble nature of the porphyrin complexes, the as-prepared aerogel catalysts were highly compatible with the aqueous phase. Their catalytic activities were tested in the mineralization reaction of phenol, 3-chlorophenol, and 2,4-dichlorophenol with hydrogen peroxide. The results show that both aerogels catalyzed the oxidation of phenol and chlorophenols to harmless short-chained carboxylic acids under neutral conditions. In batch experiments, and also in a miniature continuous-flow tubular reactor, the aerogel catalysts gradually reduced their activity, due to the slow oxidation of the porphyrin ring. However, the rate and extent of the degradation was moderate and did not exclude the possibility that the as-prepared catalysts, as well as their more stable derivatives, might find practical applications in environment protection.

## 1. Introduction

Phenol is an extensively used reagent in the production of phenolic resins, bisphenol A, caprolactam, and other chemicals. As an unfortunate consequence, similarly to many compounds used in the industry, phenol can be found in wastewater or in the soil. Due to its high toxicity, complete elimination of phenol is an important environmental goal.

Chlorophenols are produced by the chlorination of phenol, resulting in 19 different compounds including isomers. All of them show significant antiseptic activity and higher toxicity than that of phenol. That property is utilized when they are applied as disinfectants, preservative agents, herbicides, or insecticides [1]. However, these compounds are not biodegradable at all. By bioaccumulation in plant and animal species, they can affect the food chain. Due to their persistence in the environment, they are listed as first-priority pollutants, so the necessity of their effective mineralization is indisputable [2]. Since chlorinated organic compounds are resistant to biodegradation, processes other than microbial processes are needed to eliminate them [3]. Thus far, many techniques have been developed for the removal of phenols from the environment [4,5]. The most efficient methods are the advanced oxidation processes (AOPs), in which hydroxyl or sulfate radicals act as active agents. In practice, several reactions and materials are used to generate them, such as UV light combined with either hydrogen peroxide or ozone, the Fenton reaction, or salts of the peroxy monosulfate ion, for example [6,7,8,9].

In the Fenton and Fenton-like reactions, hydroxyl radical (OH^−^) is generated from hydrogen peroxide, although the exact mechanism is still not clarified. H_2_O_2_ can oxidize Fe^2+^ ion to produce Fe^3+^ ion, hydroxide ion and highly reactive radicals (see Equations (1) and (2)) and these free radicals can easily oxidize the organic pollutants [10,11,12].

(1) $${\mathrm{Fe}}^{2+}+H_{2}O_{2}\;\to {\;\mathrm{Fe}}^{3+}+{\;\mathrm{OH}}^{-}+{\;\mathrm{OH}}^{•}$$

(2) $${\mathrm{OH}}^{•}+{\;H}_{2}O_{2}\;\to {\;\mathrm{HO}}_{2}^{•}+{\;H}_{2}O$$

The Fenton-like metal ion catalysts are used mostly in the homogeneous phase. Their main drawbacks are the difficulty of separation and regeneration of the catalyst, as well as the increasing concentration of metal ions in water and the soil.

The most common catalytically active materials are metals, oxides and sulfides. The efficiency of the heterogeneous catalysts may be increased by applying them on a solid support. Although the turnover rate is in general lower compared to the homogeneous phase reactions, heterogeneous catalysis has several advantages. Easy separation from the reaction mixture, better tolerance towards extreme reaction parameters, larger surface area and a higher number of active sites make them valuable materials [13,14].

Numerous heterogeneous catalysts have been studied, such as, the Cu- or Fe-containing zeolites due to their high catalytic activity and selectivity [15,16,17]. Additional examples are the porphyrins, which are tetradentate macrocyclic ligands and form complexes of extremely high stability. Various porphyrin derivatives containing zinc, copper, iron, manganese, palladium, vanadium or other transition metal ions are extensively used as catalysts [18,19,20]. In the homogeneous phase catalytic processes, porphyrins are susceptible to self-degradation and loss of activity [21,22]. Therefore, it is an important goal to develop heterogeneous phase porphyrin catalysts possessing high activity and increased stability.

Aerogels are extremely light solid materials exhibiting unique physical and surface properties. Some of the most versatile ones are silica aerogels, which are prepared by the sol-gel technique from an organic silane precursor, and dried to a solid under supercritical conditions. The process results in a substance with specific properties, such as high and open porosity, large specific surface area, extremely low bulk density, high insulating capacity, to name a few. Thanks to these features, aerogels can be applied in many different industrial fields, for example as insulating materials, Cherenkov radiators, biomaterials, or catalysts [23,24,25,26,27]. The siloxane network can be functionalized by covalently binding organic moieties to the skeleton, embedding guest particles in the structure, or adsorbing metal ions on the surface. Such aerogel-based materials are widely used as catalysts in hydrogen production [28,29], dye degradation in wastewaters [30], or methanol electrooxidation [31,32].

There are several methods for the immobilization of porphyrins on a carrier. The simplest technique is adsorption of the molecules on the surface [33]. Although the process is straightforward, the chance of leaching from the matrix is rather high. Another method is the “ship in a bottle” process [34], which embeds the molecules in narrow necked cavities. A major disadvantage of such a catalyst is the limited access of substrates to the catalytically active centers. The most sophisticated way is the covalent immobilization, which prevents leaching from the solid phase, and provides a good contact with the substrates. However, the technique may require special knowledge of synthetic chemistry [35,36,37].

In an earlier study it was demonstrated that highly water-soluble porphyrin complexes may undergo decomposition in Fenton reactions due to their autocatalytic oxidation [38]. It was supposed that immobilization of catalytically active porphyrin complexes may decrease the rate of autooxidation. Recently, silica aerogels covalently functionalized with tetraaza macrocyclic copper complexes were prepared and tested in our laboratory for catalytic oxidation of phenols with hydrogen peroxide [26]. In this paper we report the synthesis and characterization of silica aerogels which are covalently functionalized with water-insoluble 5,10,15,20-tetrakis(4-aminophenyl)porphyrin complexes. Their catalytic activities were tested in mineralization of environment-polluting materials, phenol and chlorophenols, in batch mode and in continuous-flow microreactor and the life expectances of the catalysts were determined at different temperatures and molar ratios.

## 2. Results and Discussion

### 2.1. Preparation of the Heterogeneous Catalysts

**Scheme 1:**
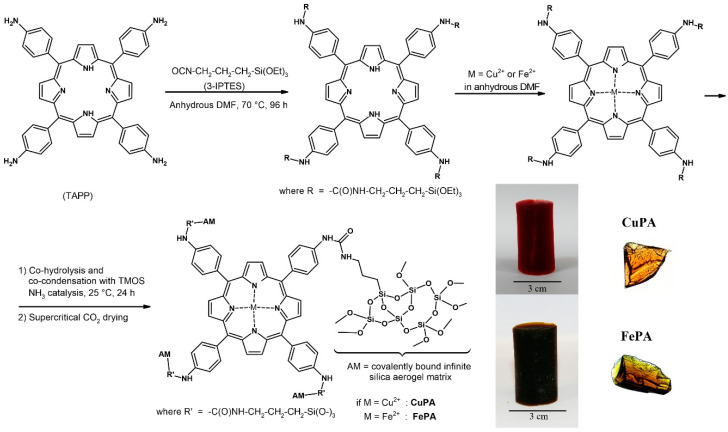
Synthetic pathway and reaction conditions of the complex formation, covalent binding and co-gelation of the porphyrin complexes with tetramethoxysilane (TMOS) leading to hybrid metal complex–aerogel catalysts FePA and CuPA. On the right side: Photographs (left) and microscopic images (40×) (right) of monolithic and fragmented pieces of CuPA and FePA catalysts.

Scheme 1 shows the synthetic steps of the preparation. First, the porphyrin ring was functionalized with 3-isocyanatopropyl triethoxysilane (3-IPTES), which acted as a bifunctional spacer and coupling agent. A 40.4 mg (5.99 × 10^−5^ mmol) portion of TAPP was dissolved in 8.00 mL anhydrous DMF under an argon atmosphere. To that 0.67 cm^3^ (656 mg, 2.39 × 10^−3^ mmol) of 3-IPTES was added, and the solution was heated at 70 °C for 96 h. Metal complexes were prepared then by carefully reacting the functionalized porphyrin rings with substoichiometric portions of either copper(2+) acetate or crystalline iron(2+) sulfate heptahydrate under anhydrous conditions in DMF or DMSO until the UV fluorescence of the free porphyrine ring disappeared. The aerogel catalysts were obtained by the ammonia-catalyzed co-hydrolysis and co-condensation of the triethoxypropylsilyl-functionalized porphyrin complexes with tetramethoxysilane (TMOS) in a methanol-water mixture using the sol-gel technique, as described earlier. After the necessary ageing and solvent exchange process, the supercritical carbon dioxide drying process resulted in aerogel monoliths [39]. The photographs of the as-prepared catalysts are shown in Scheme 1. Although it might be difficult to see their true colour in the cylindrical form, microscopic images of thin fragments revealed the red colour of the copper-containing (denoted as CuPA), and greenish brown colour of the iron-containing (denoted as FePA) aerogels.

### 2.2. Characterization of the Catalysts

The functionalization of the porphyrin ring with 3-isocyanatopropyltriethoxysilane (3-IPTES) was monitored by NMR spectroscopy (See in Figures S1 and S2). The differences between the two spectra can be clearly seen. The significant change in the aromatic region of the spectra (Figure S2) indicates that the coupling was successful. The complexation of the functionalized porphyrin ring with Cu(2+) and Fe(2+) ion was monitored with a 366 nm UV lamp. The non-complexed porphyrin ring had a strong red fluorescence, which disappears when the complex is formed. We could observe the vanishing of the fluorescence in the case of both metal ions.

**Figure 1:**
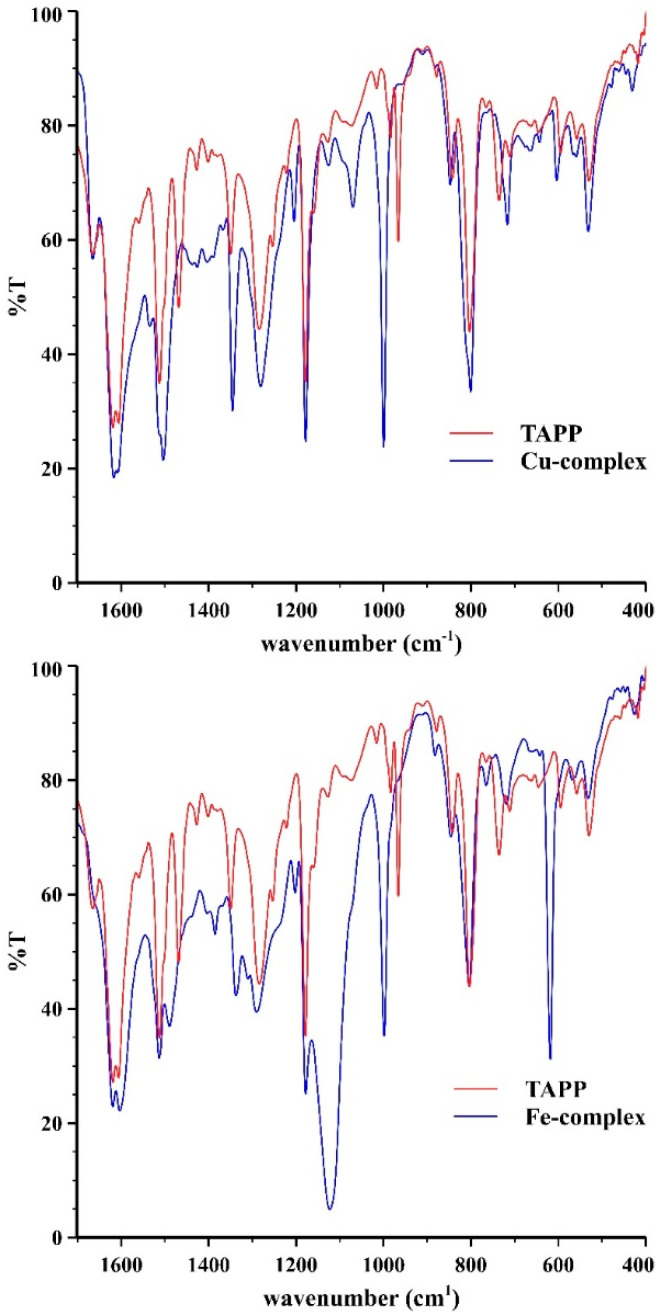
Fingerprint region of the FT-IR spectra of the free copper and iron complexes (blue curves) compared with that of the empty porphyrin ring (red curves). The full recorded spectra can be seen in the Supplementary Materials (Figure S3). Although accurate assignation of the peaks is not available, a detailed analysis of the IR spectra of tetraphenyl porphyrin complexes is available in the literature [42].

**Figure 2:**
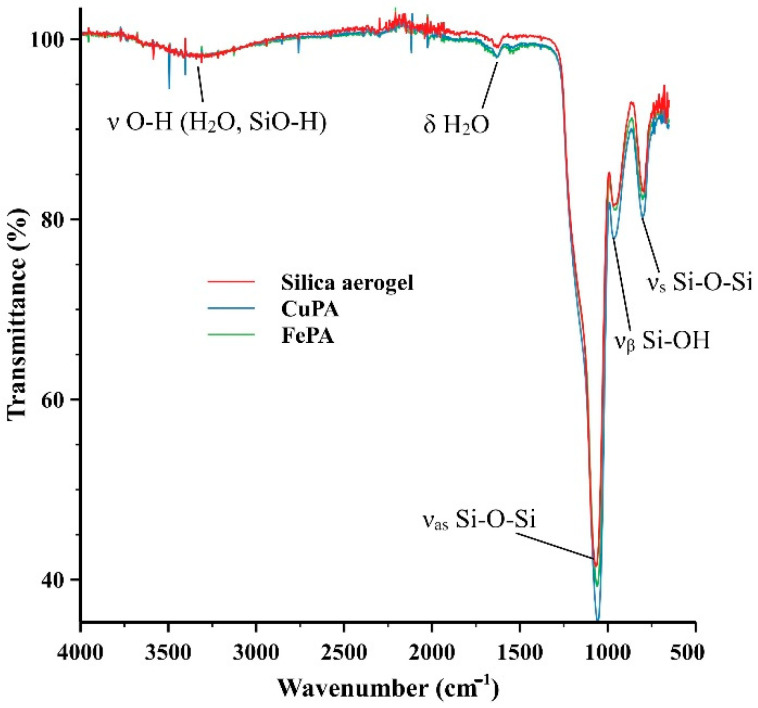
FT-IR spectra of the aerogels CuAP and FeAP functionalized with the copper and iron porphyrin complexes. They are in good agreement with the spectra published in the literature for silica aerogels [43] and silicas covalently coupled with porphyrins [44]. Due to the low concentration of the complexes in the silica aerogel matrix, the characteristic peaks of the complexes shown in Figure 1 are too weak to be observed.

The FT-IR spectra of the complex solutions were recorded as well, they can be found in the Supplementary Materials (Figure S3). Figure 1 shows the fingerprint regions of the spectra compared with that of the non-complexed porphyrin ring. The differences prove the change in the structure of the porphyrin ring and thus the formation of the complexes. Figure 2 shows the FT-IR spectra of an aerogel functionalized with porphyrin complex. The following peaks can be assigned to silica aerogels: The O–H stretching vibration at ~3400 cm^−1^, the Si–O–Si asymmetric stretching vibration at around 1050 cm^−1^ and the asymmetric stretching vibration at approximately 950 cm^−1^. The vibrations of C–H bonds appearing between 2800 and 3000 cm^−1^ indicate the successful functionalization.

**Figure 3:**
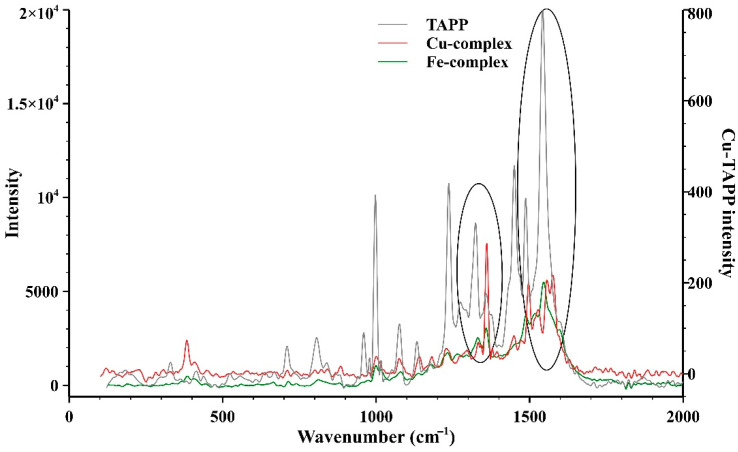
Raman-spectra of the empty 5,10,15,20-tetrakis(4-aminophenyl)porphyrin ring (TAPP) and its Cu- and Fe-complexes. In the case of Cu-complex, a different intensity scale was used for easier comparability of the spectra. The differences between the positions of the circled peaks—which can be assigned to the aromatic hydrocarbons, in which a hydrogen has been replaced by amino group—indicates change in the structure and the formation of the complexes.

**Figure 4:**
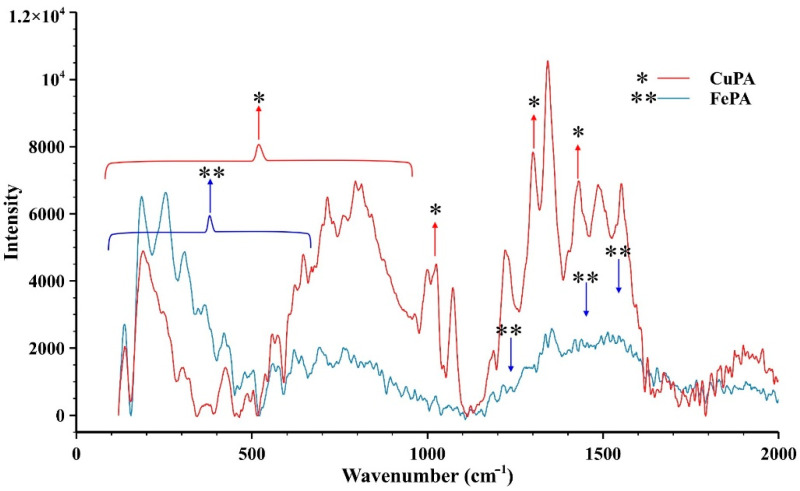
Raman-spectra of the aerogel materials CuPA and FePA. Due to the low concentration of the porphyrin complex, the spectrum of FePA is less informative than that of CuPA. The spectral differences and the direction of the intensity changes of the CuPA and FePA aerogels compared to the complexes (shown in Figure 3) clearly indicate the formation of covalent bonds between the functionalized porphyrin complexes and the silica aerogel matrix. (*) CuPA, (**) FePA.

The Raman spectra of the porphyrin ring, the complexes and the functionalized aerogels were also recorded. Figure 3 shows the difference between the spectrum of the empty porphyrin ring and the complexes. In the high-frequency region the Raman bands are sensitive to the electron density, the axial ligation and to the core size of the central metal ion. The band at 1543 cm^−1^ of the porphyrin ligand can be assigned to the C_β_C_β_ stretch, which was upshifted to 1546 cm^−1^ in the Fe and 1576 cm^−1^ in the Cu complexes, respectively. This band appears as one of the most intense bands in the spectra for the Fe and Cu complexes. The band at 1487 cm^−1^ of the porphyrin ligand could be assigned to the phenyl ring vibration, which was practically the same in the Fe complex but was shifted to 1497 cm^−1^ in the Cu complex, indicating a higher effect of the Cu ion on the phenyl at the meso-positions. The bands between 1300 cm^−1^ and 1450 cm^−1^ are most likely the out-of-phase coupled C_α_C_β_/C_α_N stretching modes. The 1323 and 1360 cm^−1^ bands of the porphyrin ligand were most probably the pyrrole quarter ring stretching and the C_α_C_β_/C_α_N stretching modes, respectively. The C_α_C_β_/C_α_N stretching appeared at 1333 cm^−1^ for both the Cu and the Fe complexes too. The pyrrole stretching was found at 1360 cm^−1^ for the Cu and Fe complexes as well. The 1236 cm^−1^ band of the 5,10,15,20-tetrakis(4-aminophenyl)-porphyrin (TAPP) and the 1230 and 1234 cm^−1^ bands of the Cu and Fe complexes were most likely attributed to the C_m_-ph stretching. The band at 1076 cm^−1^ of the porphyrin ligand was most probably the vibration of the pyrrole C_β_–H stretching, which appeared at the same wavenumber for the Cu complex and shifted to 1080 cm^−1^ for the Fe complex. The band at 997 cm^−1^ of the porphyrin ligand was most likely the vibration of pyrrole breathing and phenyl stretching, which shifted to 1001 cm^−1^ in the case of both the Cu and the Fe complexes as well. The band at 960 cm^−1^ of the porphyrin ligand was most probably the pyrrole breathing, but it could not be found in the complexes due to the exchange of the hydrogen atom with the metal ion in the N-H bonding. The band at 710 cm^−1^ was most likely the π3, phenyl mode, which was not seen in the Cu complex but shifted to 714 cm^−1^ for the Fe complex. At 384 cm^−1^ for the Cu and at 385 cm^−1^ for the Fe complexes the bands are assigned to the M-N vibration, which cannot be seen in the porphyrin ligand [40]. For the porphyrin ligand, a weak Raman band can be seen at 330 cm^−1^, which was most likely the in-plane translational motion of the pyrrole [41]. The peaks at around 1000 cm^−1^, 1340 cm^−1^ and 1580 cm^−1^ indicate the presence of the aromatic hydrocarbons in which a hydrogen has been replaced by an amino group (benzene and pyrrole). These peaks appear in the spectra of the complexes as well as in that of the empty porphyrin ring but at a slightly different position (circled peaks in the spectra). For easier comparability, adifferent intensity scale was used in the case of the Cu-porphyrin complex since the intensities were too weak compared to the other two samples. Figure 4 shows the Raman spectra of the functionalized aerogels. Since the aerogels contain a little amount of the complexes, it was quite difficult to record acceptable quality spectra. Despite this hardship, the difference between the spectrum of the CuPA aerogel and that of the free complexes clearly indicate a change in the structure of the complex by which the formation of the covalent bond between the complex and silica framework is confirmed.

The results of the elemental analysis can be seen in Table 1. The non-zero carbon and nitrogen content of the blank aerogel sample indicates that after the supercritical drying adsorbed residues of the solvents and the catalyst ammonia may be present in the aerogels. Compared to that as a background, both the carbon and nitrogen contents of CuPA and FePA are higher in the obtained samples, indicating the successful incorporation of the porphyrin complexes.

**Figure 5:**
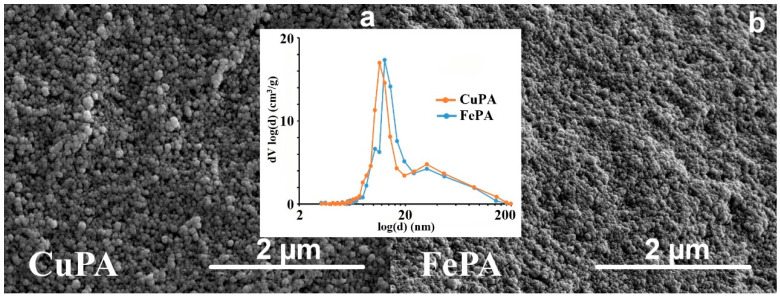
Scanning electron micrographs of the aerogel catalysts (**a**) CuPA, and (**b**) FePA. Insert: Pore size distribution curves calculated by the BJH method for CuPA (red line) and FePA (blue line). Both of the curves show the characteristic pore diameter of around 12 nm, which is typical for the silica aerogels.

Porosity of the aerogel samples was measured by nitrogen adsorption porosimetry at 77 K temperature, after degassing the samples at 100 °C for 24 h. Figure 5 shows the cumulative pore volumes and pore size distribution curves calculated by the BJH method. Nitrogen adsorption-desorption isotherms can be found in the Supplementary Materials (Figure S4). The specific surface area of the CuPA and FePA aerogels was 980 m^2^/g, and 1019 m^2^/g, respectively. The actual values are significantly higher than the 600–700 m^2^/g values characteristic of pristine silica aerogels prepared by the same method. Bulk densities of the functionalized aerogels were in the range of 0.076–0.085 g/cm^3^, which was a bit lower than 0.085–0.090 g/cm^3^ obtained for the pristine silica aerogels. Measured and calculated parameters are given in Table 2. The values are characteristic of the silica-based aerogels in general, thus the incorporation of the porphyrin complexes did not alter the gel structure significantly. Most of the pores are in the 2–50 nm mesopore region, and only a negligible volume falls in the less than 2 nm diameter micropore region, as calculated by the t-plot method.

Scanning electron microscopy (SEM) pictures were in good agreement with the porosimetry results. The samples showed the homogeneous structure, and the visible pores were in the higher mesopore and lower macropore region (Figure 5). The size of the globules and the pores are also characteristic of the silica-based aerogels. Most importantly, no detectable agglomeration of the porphyrin complexes was observed. According to these results a uniform and molecular level distribution of the complexes was obtained in the silica matrix.

The metal content of the aerogels was determined by an inductively coupled plasma-optical emission spectrometry (ICP-OES) method, and the results are summarized in Table 3. The measured values are a bit higher than the values calculated from the chemical composition of the reaction mixtures, due to the leaching of short-chained partially hydrolized siloxanes in the ageing and solvent exchange steps. However, the colourless nature of the ageing solutions proved that no intense-coloured porphyrin complex was lost in the process, and their entire amount was incorporated.

### 2.3. Catalytic Activity

The catalytic activity of the samples was evaluated through the oxidation of phenol and chlorophenols with hydrogen peroxide in aqueous solutions at different temperatures. The pH of the reaction mixtures was left to change spontaneously in the course of the reactions in order to simulate the behaviour of real-life wastewaters. The main oxidation products of phenol were identified by HPLC measurements; further products and the proposed degradation pathways are published in a previous work [26]. In the case of chlorinated phenols, the oxidation products were undetectable by UV/HPLC, and mass spectrometry was used instead.

We made several attempts to determine the catalytic activities of the complexes in the homogeneous phase. Unfortunately, all of them failed, due to the insolubility of the porphyrin complexes in water, water–DMF and water–DMSO mixtures. The reaction mixture seemed to be homogeneous at the applied 90 °C, nevertheless when it started to cool down, solid particles appeared and the solution turned colourless (Figure S5). The reaction could have been tested in a homogeneous phase using DMF as the solvent, in which only the calculated volume of 30% (m/m) aqueous hydrogen peroxide was dissolved. However, none of the complexes showed any catalytic activity against the phenols. The reason for this can be either that a fast self-oxidation destroyed all the porphyrin complexes, or that the oxidation of the solvent DMF, which was present in large excess, consumed the oxidant.

#### 2.3.1. Phenol Oxidation

**Figure 6:**
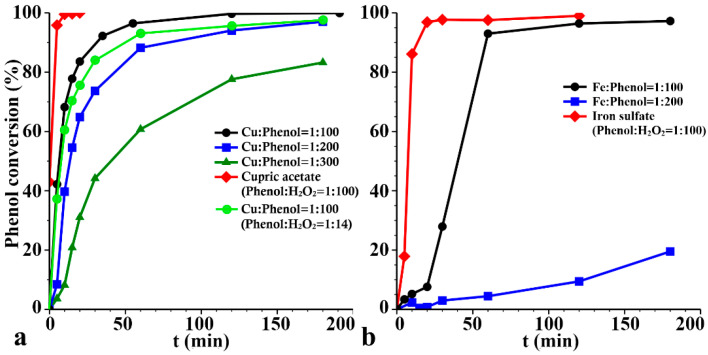
Kinetic curves of phenol conversion over time using the CuPA catalyst (**a**) and FePA catalyst (**b**), and the free metal ions using different catalyst-to-phenol (1:100, 1:200, 1:300), and phenol-to-H_2_O_2_ molar ratios (1:100 and 1:14).

Phenol oxidation and conversion was monitored by a reversed-phase HPLC technique, the concentrations were determined by using a five-point calibration curve and UV detection. Several catalyst-to-substrate, and catalyst-to-hydrogen peroxide molar ratios were tested in batch experiments. Conversion curves of phenol are shown in Figure 6. The main feature is that 80% of the phenol was converted within three hours even when the catalyst was applied only in a 0.33 mol%. Obviously, the free copper(II) ions in the homogeneous phase were much more effective. Nevertheless, in a homogeneous phase the separation of the catalyst Cu^2+^ would be difficult and it is not favorable in industrial use. As expected, the free iron(2+) ions were more effective catalysts in the homogeneous phase than the complex in the heterogeneous phase. However, when the catalyst was applied in 1 mol% quantity, more than 90% of the phenol was eliminated within one hour (Figure 6b). The FePA catalyst applied in a smaller amount did not show as high catalytic activity as the catalyst CuPA did. Both catalysts showed slightly S-shaped conversion curves, which may be the indication of autocatalytic reactions or the consequence of hindered diffusion and materials transport in the pores.

**Figure 7:**
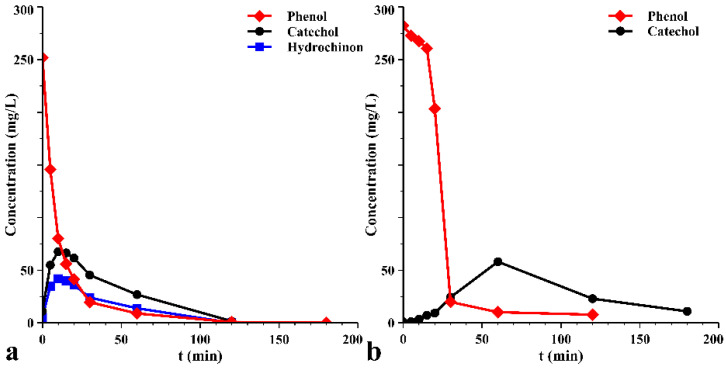
Intermediates profile of phenol oxidation by CuPA (**a**) and FePA (**b**) catalysts. In the case of the copper complex, catechol and hydrochinon were detected, and only catechol was observed when iron ions were applied.

Depending on the applied metal complexes, a different intermediate profile was obtained (Figure 7). In the case of the copper complex, two main intermediates were detected: catechol and hydrochinon. Only catechol was detected as an intermediate when the iron complex was applied, supposedly due to the different reaction mechanisms. It was confirmed by mass spectrometry that the intermediates continued to transform into further products, and finally into short-chained carboxylic acids [45].

**Figure 8:**
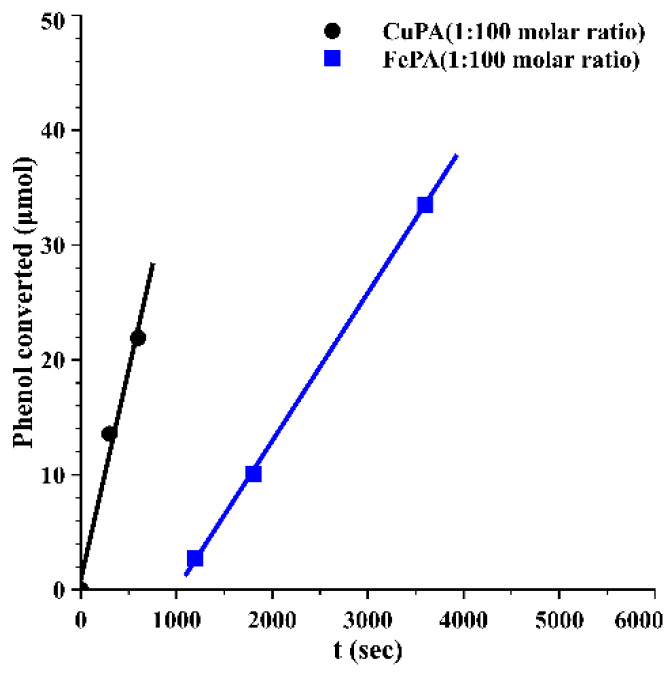
Linear trend lines fitted to points selected from the initial linear phase of the kinetic curves of phenol oxidation using CuPA (●) and FePA (■) catalysts.

In order to compare the efficiency of the catalysts more expressively, the turnover frequencies (TOFs) were calculated. We selected a set of points from the initial linear section of the kinetic curves and applied linear regression (Figure 8). The TOF values and the regression coefficients of the fittings are shown in Table 4. As it can be seen, the rate of the phenol degradation is higher in the case of catalyst CuPA but both of the TOF values are comparable to that of the industrial catalysts, since for the most relevant industrial applications the TOF values are in the range of 10^−2^–10^2^ s^−1^ [46].

Unfortunately, the catalysts lost their activity after the first cycle, most likely because the porphyrin ring suffered self-oxidation. Due to the static conditions applied during the reactions, the high excess of hydrogen peroxide could cause the oxidation of the porphyrin ring.

**Figure 9:**
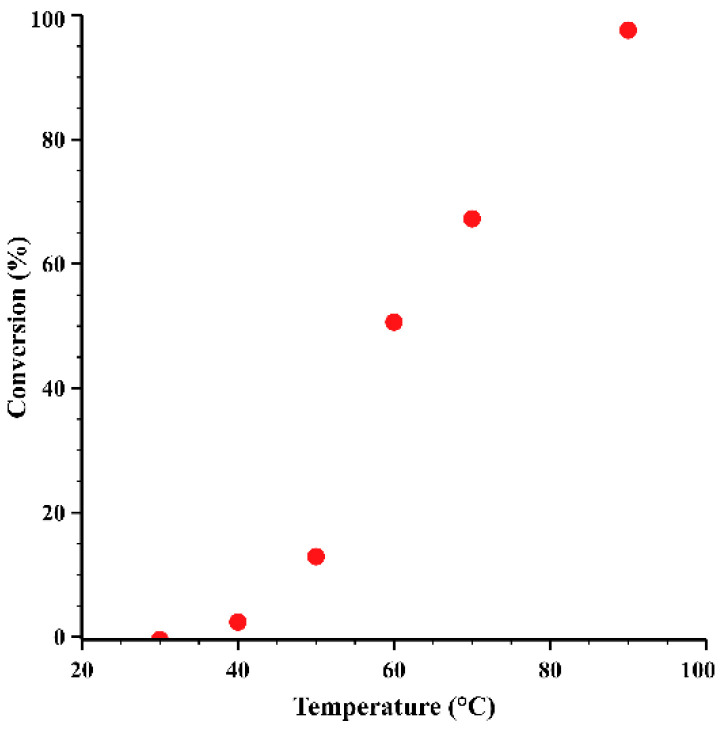
Phenol conversion after 180 min as a function of the reaction temperature. The highest conversion was achieved at 90 °C. Nevertheless, at 60 °C the degradation of the catalyst was minimal, so this value is found to be the most advantageous to carry out the reaction.

The reaction was carried out with lower H_2_O_2_ excess, the applied molar ratios were: catalyst:phenol:H_2_O_2_ = 1:100:1400. The kinetic curve can be seen in Figure 6. The conversion was higher than 90% after one hour in this case as well, but the colour of the catalyst changed this time too, which indicated the degradation of it. Therefore, we tried to optimize the temperature too. The reaction was carried out at different temperatures besides 90 °C: 30–70 °C using the catalyst:phenol:H_2_O_2_ = 1:100:1400 molar ratio. The final phenol conversion as a function of the temperature can be seen in Figure 9. We found that the highest conversion was achieved at 90 °C. There is a breakpoint between 50 °C and 60 °C, the conversion was 50% compared to 12% at 50 °C. At 60 °C the degradation of the catalyst was minimal, although the rate of the reaction was much lower than at 90 °C, the calculated turnover frequency was 4.06 × 10^−4^ s^−1^.

The quantity of the free metal ions in the reaction mixture after the decomposition of phenol was determined by an ICP-OES method. After three hours reaction time, 36% of the total copper ion, and 89% of the total iron ion was free. In a control experiment, when only the catalysts were suspended in distilled water and heated for three hours, only 3.7% of the total copper-ion was measured in the supernatant. This was in good agreement with the increasing solubility of the amorphous silica aerogel in water at elevated temperatures. The concentration of the free iron ions in the control experiment was under the limit of detection (LOD). Both experiments proved that the presence of the oxidizing agent hydrogen peroxide was the prerequisite for the degradation of the porphyrin rings, which led then to the release of metal ions. That is the reason why all of the regeneration attempts failed. The degradation of the porphyrin ring was discernible through the colour change of the catalysts. It gradually turned from red to brown in the case of CuPA, and the brown colour of the FePA catalyst slowly disappeared during the reaction.

**Figure 10:**
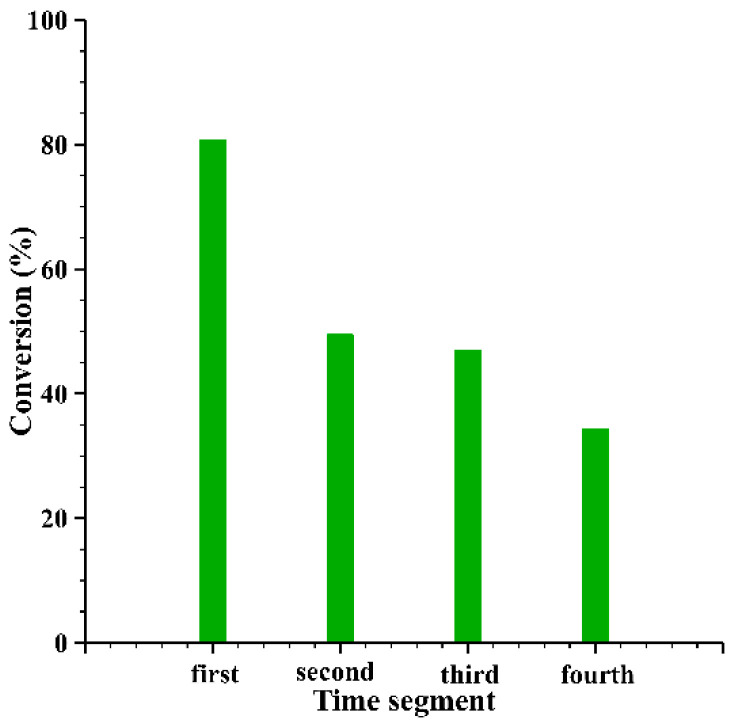
Conversion of phenol in a short-bed continuous-flow tubular reactor determined for 30 min time segments. (temperature 90 °C).

Beyond the batch experiments, a custom made continuous-flow reactor (see in Supplementary Materials, Figure S6) was tested as well. Despite of the short contact time (9 min) we observed good catalytic activity, although the efficiency gradually decreased as the reaction proceeded (Figure 10). However, it might be approaching a constant value in longer times when the neighboring catalytic centers became so distant that they could not oxidize each other. The results proved that our aerogel-based materials can be used as catalyst beds in continuous flow processes, which is more advantageous and manageable for the industry.

#### 2.3.2. Oxidation of 3-Chloro- and 2,4-Dichlorophenol

**Figure 11:**
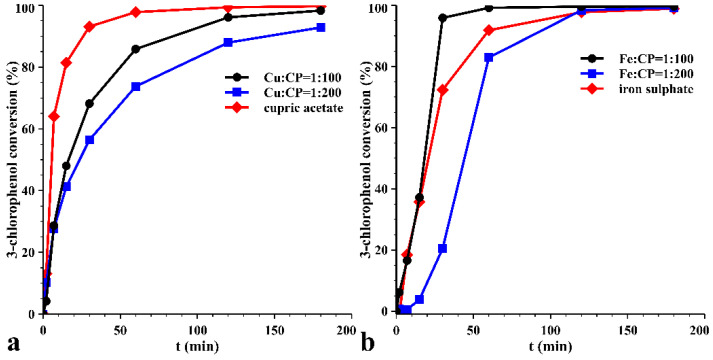
Kinetic curves of 3-chlorophenol conversion over time using the CuPA catalyst (**a**) and FePA catalyst (**b**) in different catalyst-to-phenol molar ratios (1:100 and 1:200), as well as free metal ions. The 3-chlorophenol-to-hydrogen peroxide ratio was at a constant value of 1:100.

**Figure 12:**
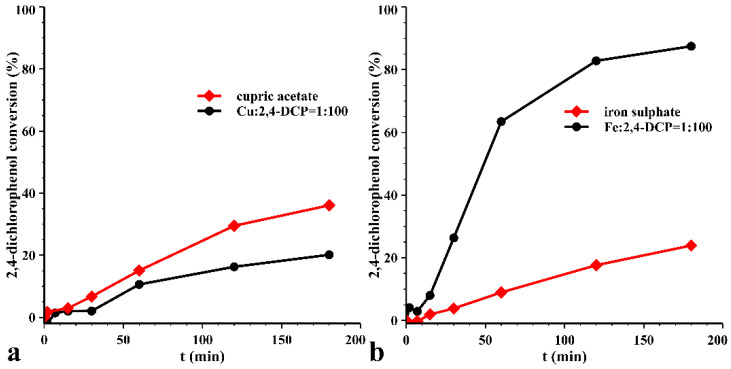
Kinetic curves of 2,4-dichlorophenol (2,4-DCP) conversion over time using the CuPA catalyst (**a**) and FePA catalyst (**b**), as well as free metal ions. The 2,4-dichlorophenol-to-hydrogen peroxide ratio was kept at a constant value of 1:100.

**Scheme 2:**
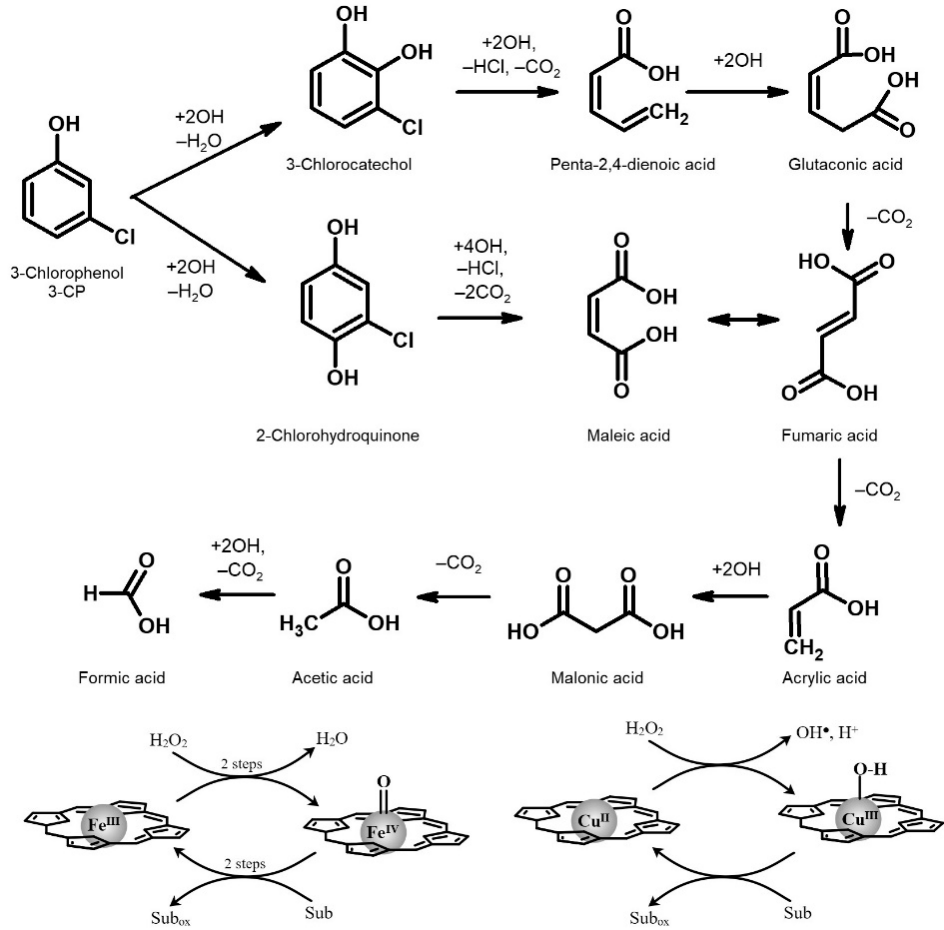
Proposed reaction pathway, formal stoichiometry, and reaction mechanisms of Fenton-oxidation of 3-chlorophenol. Through catalytic hydroxylation and dechlorination, chlorophenol was transformed into non-toxic short-chained carboxylic acids. All of the intermediates shown were identified by high-resolution mass spectrometry (HRMS).

The kinetic curves of the conversion of 3-chlorophenol (3-CP) can be seen in Figure 11. In contrast to the phenol oxidation, the catalytic activity of the CuPA catalyst was smaller than that of the FePA catalyst. Furthermore, the immobilized complex showed higher activity than the free iron ions even when it was applied in Fe:3-CP = 1:200 molar ratio. The decreased activity of the free iron ions is most likely due to the hydrolysis of Fe^3+^ ions forming catalytically inactive hydroxo-iron precipitates under the solution pH. The “S” shape of the curve in that case may also indicate either autocatalytic or diffusion controlled processes. The oxidation products were identified by high-resolution mass spectrometry (HRMS), both in the positive and in the negative ion mode. Based on the results, we suggested a reaction pathway, which is given in Scheme 2. The aromatic ring was hydroxylated first, then dechlorinated, split open and fragmented into short-chained carboxylic acids. In each case, fragmentation occurred through the loss of carbon dioxide. In the case of 2,4-dichlorophenol (2,4-DCP) (Figure 12) a significant decrease in the catalytic activity of the CuPA catalyst was observed compared to the 3-chlorophenol. Although the activity of the FePA decreased as well, it was almost as effective as in the case of the monochlorophenol. The free iron ions showed poor efficiency due to their hydrolysis under the reaction conditions, as mentioned above. Considering the complexity of the reaction, as well as the lack of appropriate analytical standards, the details of the mechanism has not been explored.

**Figure 13:**
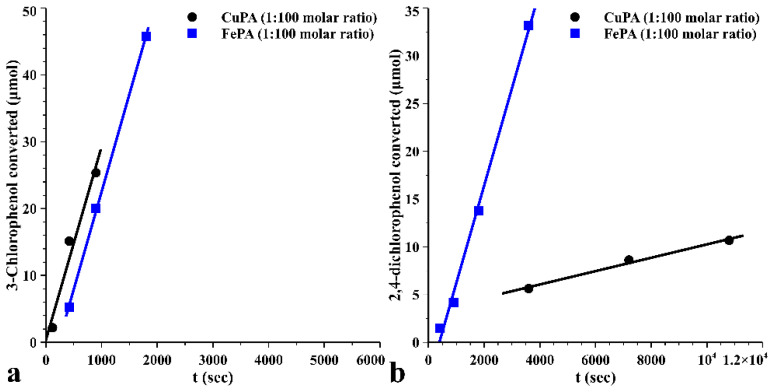
Linear trend lines fitted to points selected from the initial linear phase of the kinetic curves of 3-chlorophenol (**a**) and 2,4-dichlorophenol (**b**) oxidation using CuPA (●) and FePA (■) catalysts.

The turnover frequencies were also calculated to compare the efficiency of the catalysts (Figure 13), the results are summarized in Table 5. According to the results, the efficiency of the CuPA catalyst did not reach that of the catalysts applied in the industry if we used it for oxidation of a dichloro derivative of the phenol. In contrast to that, the TOF values of the FePA catalyst are still comparable with the industrial catalysts’ TOF values.

The chlorophenols (mono- and dichlorinated) were identified by capillary electrophoresis coupled to a mass spectrometer (CE-MS). The advantage of using this technique over HPLC is the higher sensitivity of the MS detector, which enables detection of chlorophenols in a concentration range more relevant to environmental regulations [47]. A simple method has developed for the CE separation of the chlorophenols, which also made it possible to set the limit of quantitation (LOQ) below 1 ppm, which is the regulatory limit for phenol in wastewaters. Chlorophenols are ionized more readily in the negative ion mode, therefore, the negative ion mode was applied along with separation in basic background electrolyte (40 mM ammonium formate/ammonia, pH = 9.5).

## 3. Conclusions

Copper and iron complexes of 4-aminophenylporphyrin have been immobilized successfully in silica aerogels using isocyanopropyl triehoxysilane as a bifunctional linker reagent. By functionalizing the porphyrin rings, followed by complexation with selected metal ions, we were able to bind the complexes to the silica aerogel matrix with strong covalent bonds. The as-obtained catalysts have a large specific surface area and an open mesoporous structure, which are important features of the heterogeneous catalysts.

The catalytic activity of the samples was tested through the oxidation of phenol, 3-chlorophenol and 2,4-dichlorophenol by hydrogen peroxide. The oxidation products were identified by high-pressure liquid chromatography in the case of phenol and by high-resolution mass spectrometry in the case of 3-chlorophenol. All intermediates of the degradation process were identified and a reaction scheme was proposed to describe the entire process. The FePA and CuPA catalysts showed different selectivity towards the substrates. In the case of phenol, the copper complex proved to be more efficient, while for the chlorinated derivatives the iron complex showed significantly higher activity. We have demonstrated that they can be used both in the batch and the continuous-flow modes.

Both the CuPA and FePA catalyst oxidized the dangerous phenolic pollutants in water by the safe agent hydrogen peroxide. Phenol, 3-chlorophenol and 2,4-dichlorophenol were converted to non-toxic short-chained carboxylic acids, and then finally mineralized into carbon dioxide, water and hydrochloric acid in the process. A special advantage of the process is that it can be used directly with contaminated natural waters, as the process does not require any buffering or use of additives.

Our study clearly showed that the covalent incorporation of the ab ovo water-insoluble porphyrin complexes in a hydrophilic silica aerogel matrix made them compatible with the aqueous medium and allowed their active use in such an environment. By immobilization, the extent and the rate of the catalysts’ self-oxidation was reduced, although it was still present at a lower level. Since the direct interaction of the catalytically active centers of the complexes was not possible due to their immobilization, the degradation was most likely the consequence of the attack of active hydroxyl radicals generated in the catalytic cycles. For future application, the chemical stability of the complexes should be improved for example by changing the nature of the connecting pendant arms [48]. In order to minimize the degradation of the catalysts but keep the reaction fast enough, the temperature was optimized to 60 °C and the phenol:hydrogen peroxide molar ratio was cut back to 1:14, which is close to the theoretical limit of 1:12–14 molar ratio required for complete mineralization of chlorinated phenols. However, under such conditions, the turnover frequency dropped from the 10^−1^–10^−2^ s^−1^ range to as low as 4.1 × 10^−4^ s^−1^. Our results show that the as-obtained catalysts CuPA and FePA may be considered as potential alternatives for the mineralization of phenols with the environmentally safe oxidizing agent hydrogen peroxide.

## 4. Materials and Methods

### 4.1. Materials

Methanol (technical grade), acetone (technical grade), 30% hydrogen peroxide solution (analytical reagent grade), N,N-dimethylformamide (DMF) (analytical reagent grade) and 25 m/m% ammonia solution (analytical reagent grade) were purchased from Molar Chemicals Kft. (Hungary). Tetramethyl orthosilicate (TMOS) (purum), 3-chlorophenol (98 %) and 2,4-dichlorophenol (99 %) were obtained from Sigma-Aldrich Ltd. (St. Louis, MO, USA). 5,10,15,20-Tetrakis(4-aminophenyl)porphyrin (TAPP) and 3-isocyanatopropyltriethoxysilane (3-IPTES) were purchased from ABCR GmbH (Germany). Phenol (analytical reagent grade), copper acetate (reagent grade) and iron(II) sulfate (reagent grade) were acquired from Reanal Finomvegyszergyár Zrt. (Hungary). Carbon dioxide cylinder was purchased from Linde Gáz Magyarország Zrt. (Debrecen, Hungary). All the reagents were used without any further purification.

### 4.2. Synthesis of Porphyrin Complexes

The synthesis of the complexes was carried out in two consecutive steps. First, the porphyrin ring was functionalized with 3-isocyanatopropyltriethoxysilane (3-IPTES) in a two neck flask under anhydrous conditions. A total of 20 mg of 5,10,15,20-tetrakis(4-aminophenyl)porphyrin (2.97 × 10^−2^ mmol) was dissolved in 4.0 mL anhydrous DMF and kept under a dry argon atmosphere. 0.33 mL (1.191 mmol) of 3-IPTES was added to the solution also under an argon atmosphere. The mixture was stirred at 70 °C for five days. Next, the complexes were obtained by mixing 1.0 mL of 3-IPTES-TAPP with either copper or iron salts. The reaction mixtures were diluted to 4.0 mL final volume with DMF and stirred at 116 °C in sealed vessels. The metal ions were dosed in small portions until the red fluorescence of the free base 3-IPTES-TAPP disappeared, indicating the complete formation of the complexes. The reaction time depended on the metal-ions. The Cu^2+^-TAPP-3-IPTES complex formed typically in 10 min, while the formation of the complex with iron ion took up to 72 h. The progress of the reactions was monitored by TLC.

### 4.3. Synthesis of Heterogeneous Catalysts

The catalysts (denoted as CuPA and FePA) were obtained by covalently binding the complexes to the silica precursors and then co-gelated with TMOS to develop the hybrid aerogel matrix. Two monoliths were prepared via the following general recipe. Two solutions (labelled “A” and “B”) were prepared. Solution “A” contained 12.0 mL MeOH (297 mmol) and 3.0 mL TMOS (20.33 mmol). Solution “B” was made from 12.0 mL MeOH (297 mmol), 2.00 mL of the complex solution (3.705 × 10^−3^ mmol), 1.0 mL distilled water (5.54 mmol) and 1.00 mL of diluted (1:1) NH_3_ solution (7.34 mmol). Solutions “A” and “B” were mixed together, then poured into cylindrical plastic molds (66 × 28 mm) and sealed with parafilm. The gels were kept in the molds overnight then they were transferred into perforated aluminum frames. The frames provided mechanical support and allowed for quick and efficient exchange of the solvents before supercritical drying. All gels were soaked in the following solvents in order to purify them and to remove the water: methanol-cc NH_3_ (8:1), pure methanol; each for a day. Then methanol was gradually replaced by acetone, changed in 25% steps. There was no indication of leaching of the porphyrin complexes during the process. Finally, the gels were stored in a copious volume (2 L) of freshly distilled dry acetone for three days. After the change of the solvents, the aerogels were obtained by supercritical drying, which was carried out in a custom-made high-pressure reactor according to a general procedure published in a previous work [39].

### 4.4. Characterization of the Catalysts

Nitrogen gas porosimetry measurements were performed on a Quantachrome Nova 2200e surface area and porosity analyzer (Quantachrome Instruments, Boynton Beach, FL, USA). Pieces of the samples were ground in a mortar and outgassed under vacuum at 100 °C for 24 h before the measurements.

1H-NMR measurements were performed on a Bruker 360 spectrometer (Bruker Billerica, MA, USA).

FT-IR spectra were recorded on a Jasco FT/IR-4100 instrument (Easton, MD, USA).

Raman measurements were performed on a Renishaw InVia Raman microscope (Renishaw, Wotton-under-Edge, United Kingdom). It was used for the characterization of the aerogel samples in the range 100–5000 Raman shift/cm^−1^. The laser used for the measurements was a 532 nm, 50 mW diode laser. All spectra were recorded at a 10 s exposure time each, utilizing 2400 L/mm grating. Beam centering and Raman spectra calibration were performed daily before spectral acquisition using the inbuilt Si standard.

Elemental analysis was performed in a varioMICRO element analyzer (Elementar Analysensysteme GmbH, Hanau, Germany).

Scanning electron micrographs (SEM) were recorded on a Hitachi S-4300 instrument (Hitachi Ltd., Tokyo, Japan) equipped with a Bruker energy dispersive X-ray spectroscope (Bruker Corporation, Billerica, MA, USA). The surfaces were covered by a sputtered gold conductive layer and a 5–15 kV accelerating voltage was used for taking high resolution pictures.

The analysis of the metal content of the aerogels was carried out by an Agilent ICP-OES 5100 SVDV device (Agilent Technologies, Santa Clara, CA, USA) after nitric acid and hydrogen peroxide microwave digestion.

### 4.5. Study of Catalytic Activity

The catalytic activity of the aerogels was tested through the decomposition of phenol and chlorophenols in an aqueous solution.

In the case of phenol, typically the phenol solution was placed into a flask along with the appropriate amount of catalyst; the molar ratios were catalyst to phenol 1:100, 1:200 or 1:300. The required amounts of the aerogels were calculated according to the ICP results. The mixture was thermally equilibrated for several minutes before hydrogen peroxide (molar ratios phenol to hydrogen peroxide 1:100 or 1:14) was added to start the reaction. The total volume was 15.0 mL. The initial concentration of phenol was 250 ppm. The reaction mixtures were stirred in closed vessels between 30–70 °C and at 90 °C for three hours. A total of nine samples were taken (1.0 mL each) and centrifuged. The supernatants were analyzed with HPLC, applying the following parameters: the column was a Phenomenex Phenyl Hexyl column (150 × 4.6 mm, particle size: 5 μm); the composition of the mobile phase was 50% H_2_O, 50% MeOH; the flow rate was 1.0 mL/min and the analysis time was 5 min. The components were detected by a UV detector at 270 nm.

Beyond the batch experiments, the oxidation of phenol was carried out in a continuous-flow reactor as well. The applied catalyst to phenol and phenol to hydrogen peroxide molar ratios were both 1:100. The phenol concentration was 500 ppm. The reaction mixture—including the phenol solution, water and hydrogen peroxide solution—was filled in a syringe and was dosed by a syringe pump with 2 mL/h rate. The catalyst was filled in a U-shaped glass tube of 0.3 cm inner diameter. The bed length was 4.3 cm, the contact time was set to 9 min. The reactor was immersed in a 90 °C water bath.

In the case of the chlorophenols, the chlorophenol solution was placed into a flask along with the appropriate amount of catalyst, the molar ratios were catalyst to chlorophenol 1:100. The mixture was thermally equilibrated at 70 °C for several minutes before hydrogen peroxide (molar ratio: chlorophenol: hydrogen peroxide = 1:100) was added to start the reaction. The total volume was 15.0 mL. The initial concentration of the chlorophenols was 500 ppm. The reaction mixtures were stirred in closed vessels at 70 °C for three hours. In total, eight samples were taken (1.0 mL each) and centrifuged. The supernatants were analyzed with HPLC, applying the following parameters. The column was a Supelcosil LC-18 (250 mm × 4.6 mm, particle size: 5 μm) column; the composition of the mobile phase was mixture of 50% ammonium acetate (50 mM)—50% MeOH in the case of 3-chlorophenol; and ammonium acetate:methanol = 20:80 in the case of 2,4-DCP; the flow rate was 1.0 mL/min, the analysis time was 8 min in both cases. 3-CP and 2,4-DCP was detected by a UV detector at 280 nm and 287 nm, respectively.

Oxidation products of chlorophenols were detected by high resolution mass spectrometry (maXis II UHR ESI-QTOF MS instrument, Bruker, Karlsruhe, Germany), both in the positive and in the negative ion mode. For positive mode ESI, the following parameters were used: capillary voltage: 3.5 kV, nebulizer pressure: 0.5 bar, dry gas flow rate: 4.5 L/min, temperature: 200 °C. For negative mode ESI, the following parameters were used: capillary voltage: 2.5 kV, nebulizer pressure: 0.5 bar, dry gas flow rate: 4 L/min, temperature: 200 °C. MS tuning parameters were optimized in both cases to measure the relevant m/z range for chlorophenols (and their oxidation products), which was 50–600 m/z, to generally detect any possible products.

A more sensitive (CE-)MS method was developed to separate and detect chlorophenols in low concentrations (~1 ppm). The abovementioned MS instrument was coupled to a capillary electrophoresis (7100 CE System, Agilent, Waldbronn, Germany) instrument via a coaxial CE-ESI sprayer interface (G1607B, Agilent). Sheath liquid was transferred with a 1260 Infinity II isocratic pump (Agilent). CE instrument was operated by OpenLAB CDS Chemstation software.

Parameters for the capillary zone electrophoretic separation: capillary: 90 cm × 50 µm fused silica; background electrolyte: 40 mM ammonium formate/ammonia (pH 9.5); applied voltage: 20 kV; hydrodynamic injection: 500 mbar·s; sheath liquid: iso-propanol:water = 1:1 containing 5 mM ammonia; sheath liquid flow rate: 10 µL/min. After each injection, a small amount of background electrolyte was also injected from a distinct vial (150 mbar) to eliminate carry-over effects. During electrophoresis, 35 mbar pressure was applied to the inlet buffer reservoir to decrease migration times significantly. The MS method in the negative mode was tuned according to the desired mass range (80–250 *m*/*z*), a much narrower one compared to the general detection of products, to obtain better sensitivity, although parameters for the ESI source were the same. Applied spectra rate was 3 Hz. Electropherograms were recorded by otofControl version 4.1 (build: 3.5, Bruker). Spectral background correction and internal calibration were performed on each electropherogram and peaks were integrated by Compass DataAnalysis version 4.4 (build: 200.55.2969).

Leaching of metal ion from the catalysts was studied by the ICP-OES technique after the following procedure: the remaining reaction mixture was centrifuged and the supernatant was filtered through a PTFE membrane filter (pore size: 0.45 μm), after which the solution was filled up in a volumetric flask. The concentrations were determined against standard solutions.

## Figures and Tables

**Table 1:** Elemental analysis of the hybrid aerogel samples. The increased carbon and nitrogen content indicate the presence of the porphyrin complexes in the silica matrix. The non-zero values of the blank silica sample are an indication of the presence of adsorbed organic residues and the catalyst ammonia after the supercritical CO_2_ drying process.

Sample\Element	C (%)	H (%)	N (%)
“blank” silica aerogel	0.75	1.41	0.36
CuPA	1.93	1.70	0.51
FePA	3.21	1.80	0.69

**Table 2:** Summary of the nitrogen adsorption/desorption porosimetry results. Specific surface area (S_BET_) was calculated by the multi-point BET method. Characteristic pore diameters (d), and total pore volumes (V_total_) were calculated from the isotherms by the BJH method. Mesopore and macropore volumes (V_meso_, V_macro_) were calculated from the cumulative pore volume curves for the regions 2–50 nm and above 50 nm, respectively. Micropore contribution (V_micr_) was calculated by the t-plot method.

	CuPA	FePA
S_BET_ (m^2^/g)	980	1019
d (nm)	12.8	11.4
V_total_ (cm^3^/g)	3.617	3.9185
V_macro_ (cm^3^/g)	0.3702	0.417
V_meso_ (cm^3^/g)	3.2218	3.4975
V_micro_ (cm^3^/g)	0.025	0.004

**Table 3:** Metal ion content of the catalysts measured by the ICP-OES technique.

Sample	Theoretical (*w*/*w* %)	Measured (*w*/*w* %)
CuPA	0.068	0.072 ± 0.001
FePA	0.084	0.110 ± 0.001

**Table 4:** Turnover frequencies (TOFs) of the immobilized complexes applied for oxidation of phenol.

Sample	TOF (s^−1^)	Regression Coefficient
CuPA	1.144 × 10^−1^	0.9816
FePA	4.05 × 10^−2^	0.9998

**Table 5:** Turnover frequencies (TOFs) of the immobilized complexes applied for oxidation of 3-chlorophenol and 2,4-dichlorophenol.

	3-Chlorophenol		2,4-Dichlorophenol	
	TOF (s^−1^)	Regression Coefficient	TOF (s^−1^)	Regression Coefficient
CuPA	5.0 × 10^−2^	0.9604	1.5 × 10^−3^	0.9884
FePA	5.0 × 10^−^^2^	0.9996	2.2 × 10^−2^	0.995

## Data Availability

Not applicable.

## Supplementary Materials

The following are available online at https://www.mdpi.com/article/10.3390/gels8040202/s1, Figure S1: ^1^H NMR spectra of the 5,10,15,20-tetrakis(4-aminophenyl)porphyrin and the coupling agent: 3-isocyanatopropyltriethoxysilane, Figure S2: ^1^H NMR spectra of the mixture of 5,10,15,20-tetrakis(4-aminophenyl)porphyrin and the coupling agent 3-isocyanatopropyltriethoxysilane. The spectra of the reaction mixture were recorded directly after mixing and after 96 hours reaction time. The changes—especially in the aromatic region—in the spectra indicate change in the chemical structure of the porphyrin ring, meaning that the functionalization was successful, Figure S3: FT-IR spectra of the empty porphyrin ring (a), and the complexes with Cu(II) (b), and Fe(II) ions (c), Figure S4: Nitrogen adsorption-desorption isotherms of the catalysts denoted as CuPA (left) and the iron-containing one, denoted as FePA (right). The shapes, as expected, are almost perfectly alike, Figure S5: Initially the reaction mixture seemed to be homogeneous (left), but after a while without heating solid particles of the porphyrin complex appeared (center), settled down and the solution became colorless (right), Figure S6: Drawing of the continuous-flow tubular reactor for phenol oxidation.
