# Supplementary material for: Environment-Friendly Catalytic Mineralization of Phenol and Chlorophenols with Cu- and Fe- Tetrakis(4-aminophenyl)-porphyrin—Silica Hybrid Aerogels
Source: Gels. 2022 Mar 23;8(4):202. doi: 10.3390/gels8040202 (PMC9027457; doi:10.3390/gels8040202)
Supplement: Supplementary file 1 [file gels-08-00202-s001.zip › gels-1621712-supplementary.pdf]

gels

MDPI

Supplementary Materials

Environment-Friendly Catalytic Mineralization of Phenol and Chlorophenols with Cu- and Fe- Tetrakis(4-aminophenyl)-porphyrin—Silica Hybrid Aerogels

Enikő Győri [1], Ádám Kecskeméti [1], István Fábián [1,2], Máté Szarka [3,4] and István Lázár [1,*]

[1] Department of Inorganic and Analytical Chemistry, University of Debrecen, Egyetem tér 1, H-4032 Debrecen, Hungary; gyori.eniko@science.unideb.hu (E.G.); kecskemeti.adam@science.unideb.hu (Á.K.); ifabian@science.unideb.hu (I.F.)
[2] MTA-DE Redox and Homogeneous Catalytic Reaction Mechanisms Research Group, University of Debrecen, Debrecen Egyetem tér 1 H-4032 Debrecen, Hungary
[3] Institute for Nuclear Research, Debrecen Bem tér 18/c, H-4026 Debrecen, Hungary; szarka.mate@atomki.hu (M.S.)
[4] Vitrolink Kft., Debrecen, Sárosi utca 9 H-4033 Debrecen, Hungary
* Correspondence: lazar@science.unideb.hu; Tel.: +36-52-512-900-22376

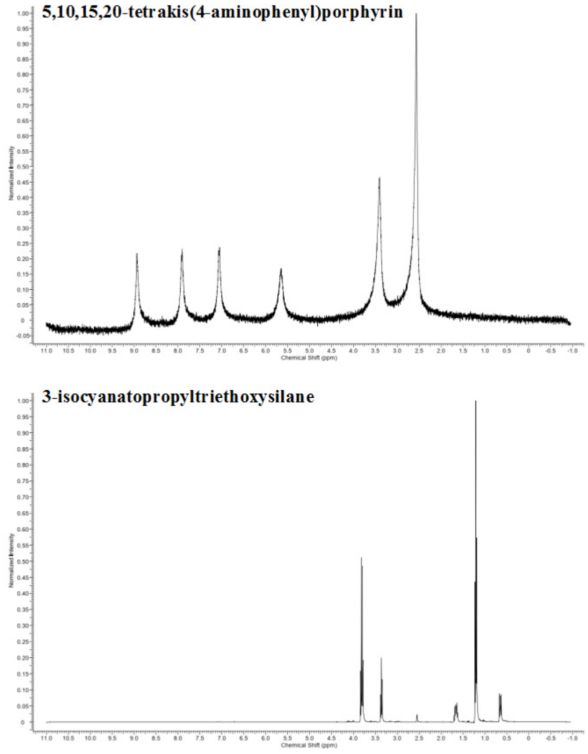

Figure S1. ^{1}H NMR spectra of the 5,10,15,20-tetrakis(4-aminophenyl)porphyrin and the coupling agent: 3-isocyanatopropyltriethoxysilane.

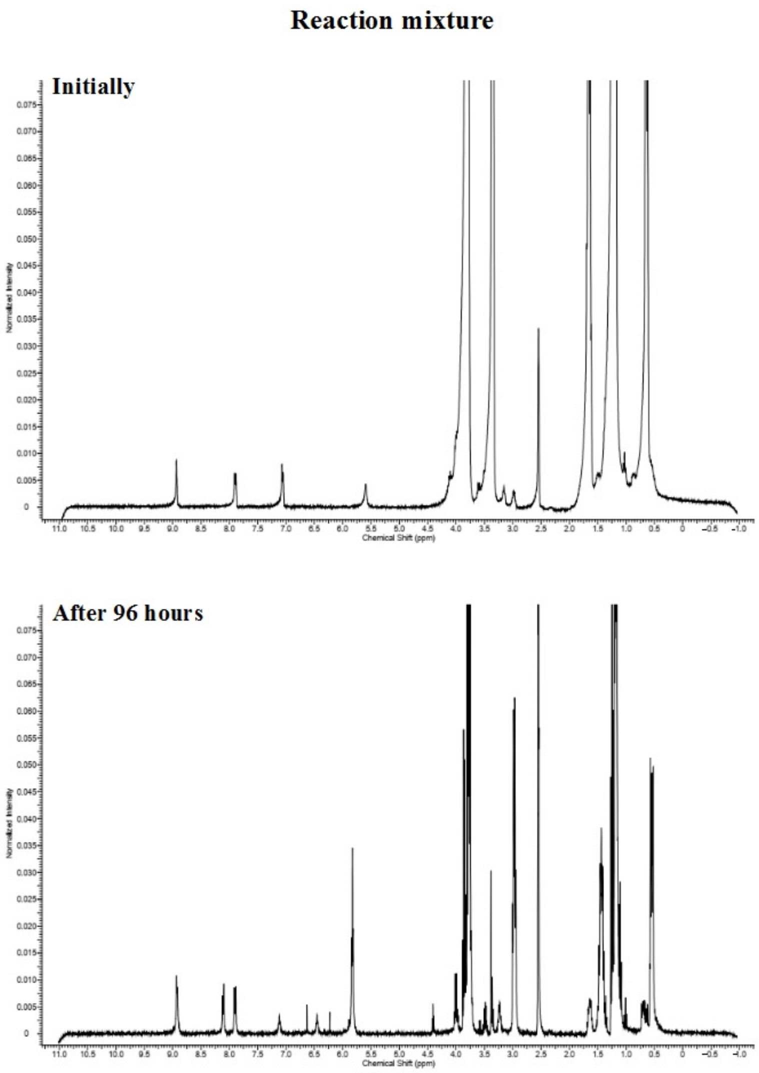

Figure S2. ^{1}H NMR spectra of the mixture of 5,10,15,20-tetrakis(4-aminophenyl)porphyrin and the coupling agent 3-isocyanatopropyltriethoxysilane. The spectra of the reaction mixture were recorded directly after mixing and after 96 hours reaction time. The changes – especially in the aromatic region – in the spectra indicate change in the chemical structure of the porphyrin ring, meaning that the functionalization was successful.

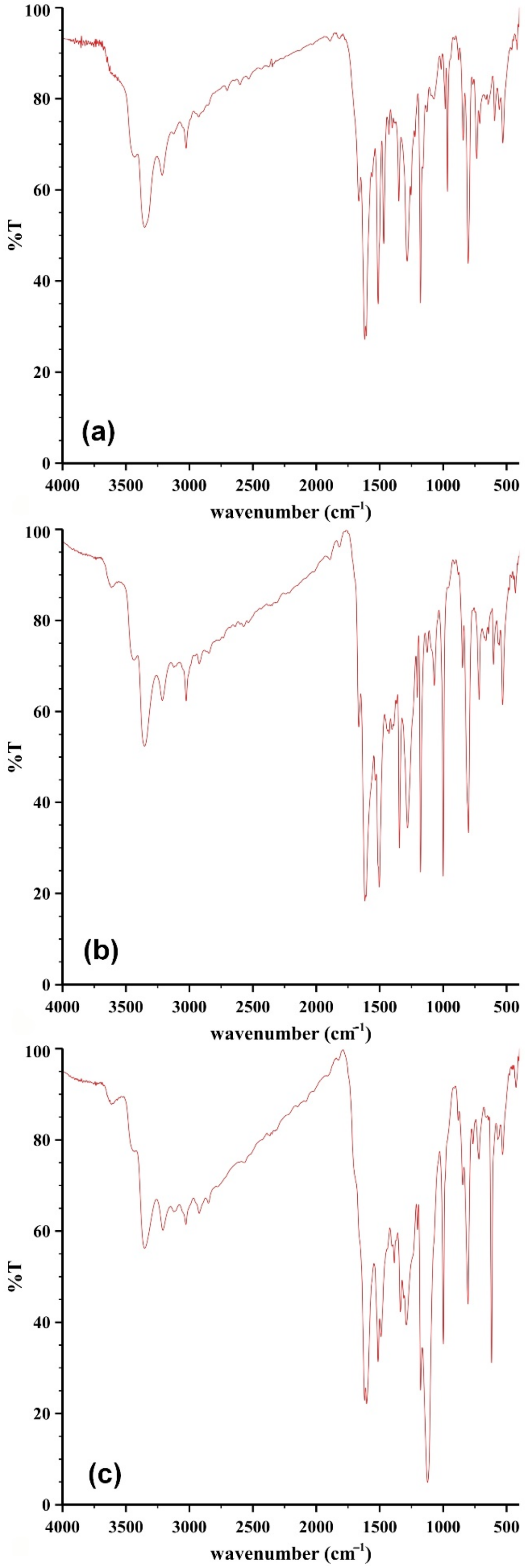

Figure S3. FT-IR spectra of the empty porphyrin ring (**a**), and the complexes with Cu(II) (**b**), and Fe(II) ions (**c**).

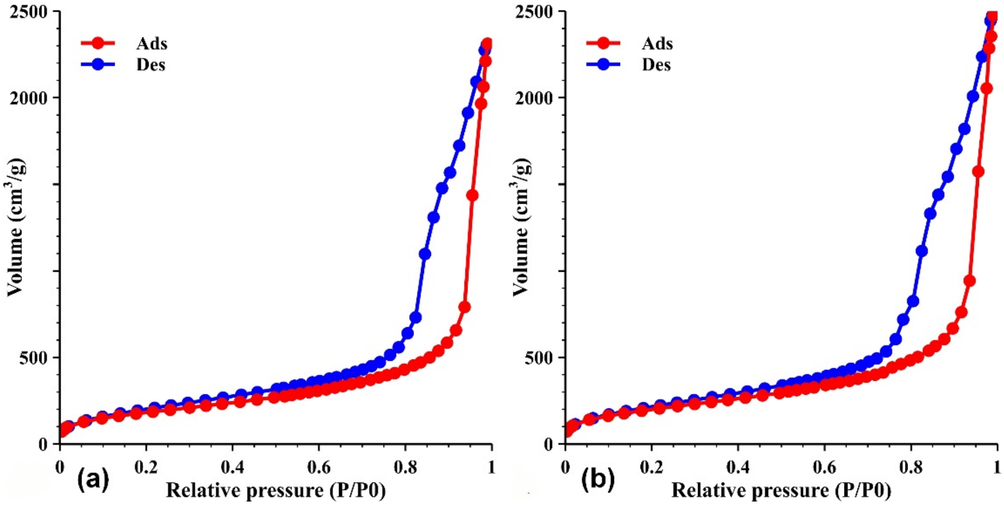

Figure S4. Nitrogen adsorption-desorption isotherms of the catalysts denoted as CuPA (**a**) and the iron-containing one, denoted as FePA (**b**). The shapes, as expected, are almost perfectly alike.

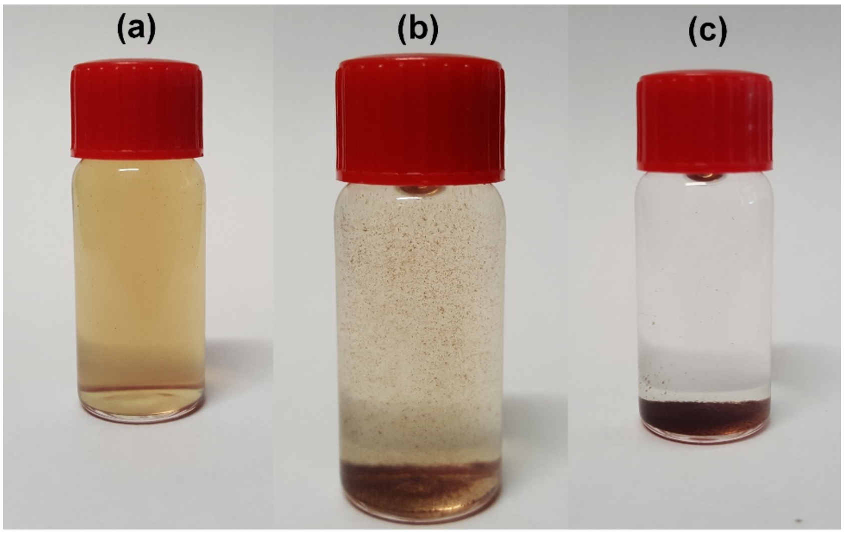

Figure S5. Initially the reaction mixture seemed to be homogeneous (**a**), but after a while without heating solid particles of the porphyrin complex appeared (**b**), settled down and the solution became colorless (**c**).

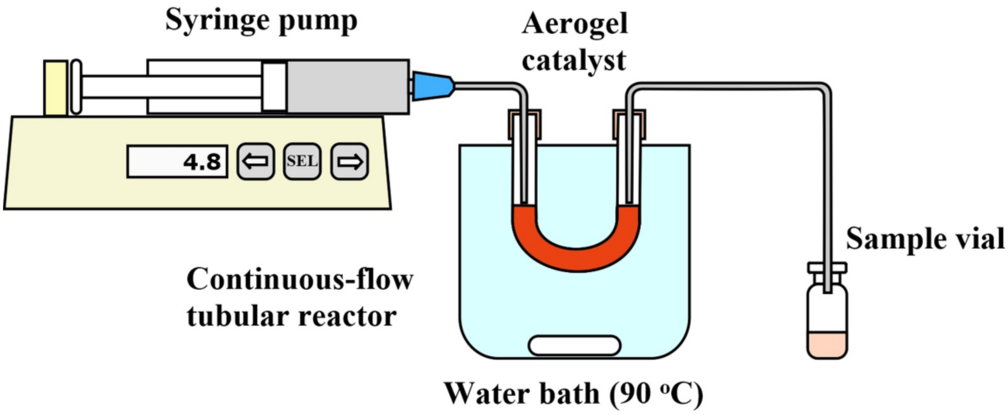

Figure S6. Drawing of the continuous-flow tubular reactor for phenol oxidation.
